# Maternal and environmental influences on egg size and juvenile life-history traits in Pacific salmon

**DOI:** 10.1002/ece3.555

**Published:** 2013-05-08

**Authors:** Douglas C Braun, David A Patterson, John D Reynolds

**Affiliations:** 1 Earth to Ocean Research Group, Department of Biological Sciences, Simon Fraser University8888 University Drive, Burnaby, British Columbia, V5A 1S6, Canada; 2 Fisheries and Oceans Canada, Cooperative Resource Management Institute, School of Resource and Environmental Management, Simon Fraser UniversityBurnaby, British Columbia, V5A 1S6, Canada

**Keywords:** Body size, fecundity, juvenile size, migration conditions, offspring size, sockeye salmon, temperature, transgenerational effects

## Abstract

Life-history traits such as fecundity and offspring size are shaped by investment trade-offs faced by mothers and mediated by environmental conditions. We use a 21-year time series for three populations of wild sockeye salmon (*Oncorhynchus nerka*) to test predictions for such trade-offs and responses to conditions faced by females during migration, and offspring during incubation. In years when their 1100 km upstream migration was challenged by high water discharges, females that reached spawning streams had invested less in gonads by producing smaller but not fewer eggs. These smaller eggs produced lighter juveniles, and this effect was further amplified in years when the incubation water was warm. This latter result suggests that there should be selection for larger eggs to compensate in populations that consistently experience warm incubation temperatures. A comparison among 16 populations, with matching migration and rearing environments but different incubation environments (i.e., separate spawning streams), confirmed this prediction; smaller females produced larger eggs for their size in warmer creeks. Taken together, these results reveal how maternal phenotype and environmental conditions can shape patterns of reproductive investment and consequently juvenile fitness-related traits within and among populations.

## Introduction

Maternal influences on juvenile life-history traits are driven by trade-offs faced by mothers and shaped by environmental conditions. The classical theory of egg size evolution suggests that female fitness is maximized by investing in egg size up to the point where offspring fitness benefits are offset by trade-offs with fecundity, such that the product of fecundity and survival per offspring is maximized (Smith and Fretwell 1974; Einum and Fleming 2000). This means that environmental conditions that influence the offspring size–fitness relationship should affect selection on egg size. Furthermore, the trade-off between egg size and fecundity can vary by female size, most commonly in species where female size influences offspring environments, such as species that build nests (Hendry et al. 2001; Hendry and Day 2003). These relationships between maternal traits, environmental conditions, and maternal fitness can be scaled up to generate hypotheses describing influences of divergent selection on egg size at the population level.

Studies of migration have provided some clear insights into how female condition, mediated by the environment, can affect reproductive investment. For example, premigratory condition and migratory duration influence laying date and clutch size of the greater snow goose (*Chen caerulescens atlantica*) (Bêty et al. 2003). Salmon in populations with more difficult migration routes produce fewer and smaller eggs for a given body size and have a higher ratio of egg number to egg size than fish with easier migrations (Fleming and Gross 1989, 1990; Kinnison et al. 2001; Crossin et al. 2004b). Furthermore, egg number shows a greater response to migration difficulty than does egg size (Kinnison et al. 2001). These findings come from comparisons among populations, but they may also apply to variation among years within populations, a concept that has not been tested.

Once females reach the breeding grounds and have laid their eggs, offspring size and the conditions the embryos face can continue to shape offspring life history and survival (Parker and Begon 1986). Larger offspring survive better in many taxa (Beacham and Murray 1985; Sinervo 1990; Fox 1994; Williams 1994; Bernardo 1996; Chambers and Leggett 1996). This bigger-is-better relationship can be mediated by environmental conditions early in life. The temperature–size rule describes a negative relationship between developmental temperatures and adult body size (Atkinson and Sibly 1997), however, this may be more complex for some organisms (Angilletta and Dunham 2003). For instance, lab experiments conducted by Beacham and Murray (1990) showed that not only was initial size strongly correlated with juvenile size but also that the largest juveniles and the highest rates of survival occurred at intermediate temperatures. Furthermore, they found that emergence timing, which is a critical life stage where juveniles switch from using their yolk for energy to feeding, was strongly determined by temperature. Higher temperatures led to faster development and earlier emergence, which could have consequences for the success of individuals during this transition from yolk to feeding. While many studies take advantage of controlled experiments and artificially propagated fish to examine the effects of egg size and environmental conditions on offspring fitness (Burt et al. 2010), there are few examples of how these interactions play out in wild populations.

Divergent selection on egg size among populations can result from differences in incubation environments that affect offspring fitness (Quinn et al. 1995; Einum et al. 2002). Theoretical (Hendry and Day 2003) and empirical (Einum et al. 2002; Rombough 2007) studies have shown that incubation environments can affect selection on egg size in salmonids. Furthermore, relationships between temperature- and juvenile fitness-related traits suggest that variation among populations in incubation temperature could be important in shaping selection on egg size among populations via differences in optimal egg sizes. While studies have tried to explain variation in egg size among populations of Pacific salmon by either demonstrating the mechanism for egg size evolution (Einum et al. 2002; Rombough 2007) or relating variation in egg size to abiotic variables (Fleming and Gross 1990; Quinn et al. 1995), none have combined these two approaches.

**Figure 1:**
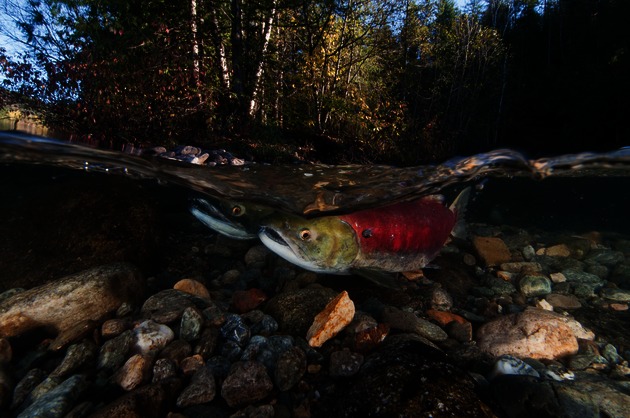
Female (foreground) and male spawning sockeye salmon on a nest in a tributary of the Fraser River. Photo: C. McCracken.

We studied 16 wild anadromous sockeye salmon (*Oncorhynchus nerka*) populations (Fig. 1), all of which experience the same long inland migrations (1100 km) to their unique spawning grounds (i.e., creeks), and similar habitats for juveniles postincubation (one or two lakes), in both freshwater and marine environments. During their freshwater migrations to their spawning grounds, adults stop feeding and use their energy reserves for migration, gonad development, and spawning. Variation in juvenile size after they hatch and emerge from the gravel where embryo development occurs can therefore be linked back to interactions between migration conditions, female size, egg size, and the conditions faced by embryos during development.

We test hypotheses for how maternal phenotype and environmental conditions influence reproductive investment in wild populations of sockeye salmon and subsequent juvenile fitness-related traits. We use both temporal and spatial analyses. The temporal analysis is based on a 21-year study of three populations, which examines migration difficulty faced by adults during their nonfeeding upstream river migration to their spawning grounds, the incubation environments of their embryos, and the size and timing of emergence of their offspring. We begin with tests of effects of interannual variation in migration difficulty (i.e., river discharge) during upstream freshwater migration to their spawning grounds on reproductive traits including egg size, egg number, total gonad mass, and the ratio of egg number to egg size. We predict that reproductive investment will scale negatively with migration difficulty and that egg number will show a greater response to migration difficulty than egg size. Using the same temporal study we then examine how variation in egg size and incubation temperatures translate into offspring fitness-related traits, including length, mass, and timing of juvenile emergence from incubation substrate. We predict that in years with warmer incubation temperatures, juveniles will be smaller and emerge earlier due to increased metabolic demands and faster development, respectively. This leads to predictions about selection on egg size, which we examine with a comparative analysis of 16 populations. We predict that populations that are generally exposed to higher temperature will produce larger mean egg sizes. We discuss our findings in the context of the interplay between maternal traits and environmental conditions mediating investment per offspring and consequences for both maternal and offspring fitness.

## Materials and Methods

### Study sites

**Figure 2:**
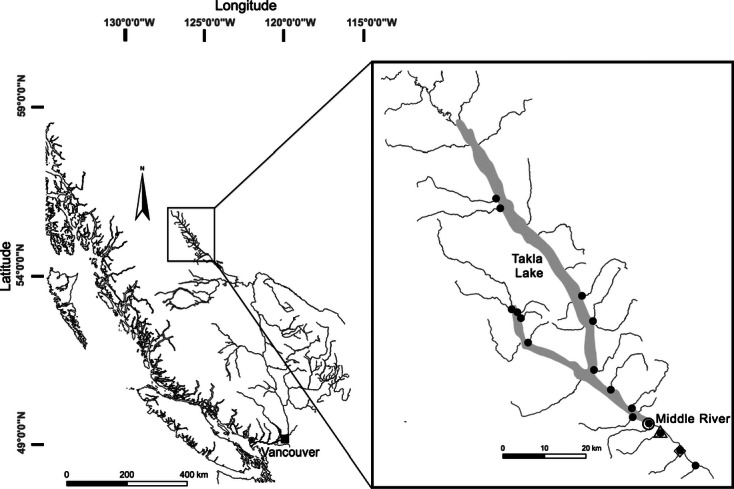
Locations of study streams • and discharge measurements ▪ in the Fraser River Basin, British Columbia, Canada. Time series data used in within-population analyses were collected for three populations (Forfar, △ Gluskie, ○ and Kynock ♢) from 1989 to 2009. Data used in among-population analyses were collected for all 16 streams in 2009.

We studied freshwater migration and spawning stream conditions for sockeye salmon populations in the Stuart drainage of the Fraser River Basin in British Columbia, Canada (Fig. 2). Sockeye salmon that migrate from the ocean to spawn in small tributaries of lakes and rivers in this drainage are known as the Early Stuart population complex. They begin their 1100 km migration up the Fraser River in early July and spawn from late July until late August. The peak of spawning occurs at similar times for all streams (within a week). Females dig nests in the gravel and bury their eggs, and juveniles emerge from the gravel the following spring. Further information on the study streams can be found in Braun and Reynolds (2011).

Our temporal dataset is a 21-year time series (1989–2009) for three populations (Fig. 2), which includes data on adult female life-history traits, migration difficulty, juvenile life-history traits, and incubation temperatures. This dataset allows tests of hypotheses for temporal variation in traits within populations. Our spatial dataset comprises 16 populations studied in 2009, including the three populations from the first dataset (Fig. 2). This provides among-population tests of relationships between female length, spawning stream temperature, and egg mass.

### Within populations: maternal length, reproductive investment, and migration difficulty

Data from the 21-year study of maternal body length and reproductive investment in three populations were collected by stock assessment personnel from Fisheries and Oceans Canada as part of annual surveys of spawning fish which are conducted to estimate annual egg deposition (Schubert and Fanos 1997). Upon arrival at the spawning grounds, unspawned females with intact ovaries (i.e., tight skeins with no signs of spawning) were sampled from each of the three study populations from 1989 to 2009 (samples per stream per year, mean = 23, range = 5–50). Fish were sampled during peak spawning, which occurs at a similar time each year. Standard body length (tip of the snout to the last vertebra in the tail) was measured to the nearest 10 mm and otoliths were removed for aging. The majority of fish sampled proved to be 4-year-olds. There were no 3-year-old females and a relatively small percentage of 5-year-old fish were found (mean = 21.3%, range = 0–87.5%).

For this dataset we used four metrics to characterize reproductive investment: (1) fecundity, (2) wet egg mass, (3) total gonad mass, and (4) the ratio of number of eggs to wet egg mass. The gonads of each fish were removed and preserved in 10% formalin. Full fecundity counts were conducted for 20% of the samples and the remaining 80% were estimated using full egg counts for a subsample of the gonads and calculated as:


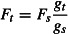
(1)

where *F*_*s*_ is the number of eggs counted from the gonad subsample, *g*_*s*_ is the mass of the gonad subsample, *g*_*t*_ is the total gonad mass, and *F*_*t*_ is the total estimated fecundity. Wet egg mass (formalin-fixed eggs) was calculated by dividing the subsample mass by the number of eggs in the subsample.

Migration difficulty was characterized by the mean Fraser River discharge over a 30-day period surrounding the 50% timing (14 July) of when fish pass through the most difficult point in their upstream migration, Hell's Gate (Macdonald et al. 2010). We also tried using the 30-day period surrounding year-specific estimates of the 50% timing date. This resulted in nearly identical results. Therefore, we continued to use the mean run timing date because it was the simpler of the two metrics. These data were collected by the Water Survey of Canada from a river gauge at Hope, BC (1989–2009), which is in the lower river just downstream of Hell's Gate (Fig. 2). We also analyzed water temperature during migration from the same location (Patterson et al. 2004). However, we do not consider temperature further in the Results for two reasons. First, temperature was highly correlated with discharge (high discharge with low temperature *r* = −0.85). Second, temperatures rarely exceeded the values that are associated with decreases in migratory performance for this particular population complex at Hells Gate (Eliason et al. 2011), as these fish usually migrate before warm temperatures become an issue.

We used linear mixed-effects regression to relate body length of females and river discharge to variation in metrics of individual reproductive investment (egg mass, number of eggs, total gonad mass, and the ratio of egg number to egg size). We also included age at maturity as a fixed factor. We did not expect relationships to vary among our three populations, but we included population as a fixed factor in all models to account for pseudoreplication. A random intercept by year was included to account for: (1) annual differences in females such as body energy condition (Crossin et al. 2004a) or disease, and (2) annual differences in the presence of fishing gear in rivers that may damage fish or impede passage (Baker and Schindler 2009), thereby influencing energy allocation to reproduction. The full model describing reproductive traits is as follows:


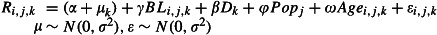
(2)

where *R* is the reproductive trait for female *i* from population *j* in year *k*, α is the intercept, γ is the effect of body length, β is the effect of discharge, ϕ is the effect of population on the intercept, and ω is the effect of age at maturity on the intercept. The residual error ε, and the year-specific variation in the intercept μ have a mean of zero and are normally distributed.

### Within populations: egg mass, incubation temperature, and juvenile fitness-related traits

Egg mass was measured as described in the previous section and juvenile data were collected by Fisheries and Oceans stock assessment personnel each spring between April and June. Juveniles are enumerated and sampled as they emerge from the stream substrate and migrate downstream toward their rearing lakes. Juveniles emerging from the incubation environment are in the fry stage, which is after they have absorbed their yolk but before they migrate to the lake. Length (to the nearest mm) and dry mass were measured for 8078 fish (juveniles per stream per year: range 100–320). Juveniles were dried at 80°C for 48 h and weighed. The number of downstream migrating juveniles is estimated using a Petersen mark–recapture method (Macdonald et al. 1992). Downstream migrating juveniles were trapped nightly using a 2 m × 3 m floating inclined plan trap and are assumed to migrate immediately after emerging from the substrate. Trap efficiency was estimated by marking 1000–2000 individuals using bismark Y biological dye and transporting them 1–2 km upstream for recapture. The estimates of downstream migrating juveniles were used to determine emergence timing, which is the date by which 50% of juveniles emerge from the substrate.

Time series data on incubation temperatures and juveniles for the three populations were available from Fisheries and Oceans Canada (Fig. 2). Temperature was measured hourly from 1989 to 2007, although some years of data were missing due to logger failure. We used daily mean temperatures to calculate three metrics that characterize incubation temperatures: (1) maximum daily mean incubation temperature (Max temp), which characterizes the warmest water temperatures experienced by incubation embryos and typically occur in August, (2) fall accumulated thermal units (ATU), which is the sum of the daily mean temperature from peak spawning to 15 November and characterizes the thermal experience for incubation embryos before temperatures drop near zero or below, usually in November, and (3) incubation ATU, which is the sum of the daily mean temperature from peak spawning to the 50% juvenile emigration date and characterizes the thermal experience of embryos throughout their development. For each juvenile trait we selected the temperature metric with the most support according to AICc relative variable importance (RVI). Details of the analyses are in Data S1 and Table S1. The temperature metrics selected are as follows: for juvenile dry mass – Max temp; and for juvenile length and 50% emigration date – Fall ATU.

We used linear regression to relate the mean population egg size and incubation temperature to mean juvenile length and dry mass at emergence, and timing of emergence. Previous studies have suggested that egg size mediates the influence of incubation conditions on juvenile size and survival (Beacham and Murray 1990; Einum and Fleming 1999); this was tested by including an egg mass by incubation temperature interaction. We also examined a population by temperature interaction to determine if relationships between temperature and juvenile traits varied by population. The full model was as follows:


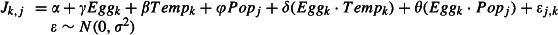
(3)

where *J* is the juvenile trait (i.e., length, dry mass, or emigration date) in year *k* for population *j*, α is the intercept, γ is the effect of egg mass, β is the effect of temperature, ϕ is the effect of population on the intercept, δ and *θ* are the effects of the interactions between egg mass and temperature and egg mass and population, and ε is the residual error.

### Among populations: maternal length, incubation temperatures, and egg mass

In 2009, females were sampled from 16 populations after they died of natural causes rather than through lethal sampling (as in the previous dataset) because of recent conservation concerns for the Early Stuart populations. This prevented us from collecting data on fecundity and total gonad mass. We measured standard length, and removed and froze any remaining eggs. Some eggs (up to 20) usually remain in the female's body cavity after they have spawned and died. Undeveloped, discolored, or broken eggs were not sampled. There are no differences in the size of eggs between those that were spawned and those retained by females. During a 3-year study, Patterson (2004) found less than 1.0 mg difference between ovulated eggs and eggs from intact ovaries (same samples used in this study). Eggs were then dried at 60°C for 48 h (Patterson 2004), weighed to one-hundredth of a milligram, and the mean dry egg mass for each female was calculated. We collected egg samples from 315 females, but were only able to obtain sufficient samples (≥10) from 179 females (females per stream: range 7–17). Preliminary analyses in which we resampled egg size from five females with ≥25 eggs showed that this sample size was adequate to encompass variation in egg sizes within females.

When testing the hypothesis that warmer incubation temperature selects for larger egg size we accounted for maternal length because longer females produce larger eggs (see Results). We compared models that included all combinations of body length, maximum mean incubation temperatures, and a body length by maximum temperature interaction to explain dry egg size using linear mixed-effects models. We used maximum mean incubation temperature as our temperature metric because it is simple, it provided the largest contrast in temperatures among populations, and it occurs during a critical stage of embryo development. Population was included as a random intercept to account for pseudoreplication. The full model for describing egg mass among populations is as follows:


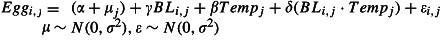


where *Egg* is the dry egg mass for female *i* from population *j*, γ is the effect of body length, β is the effect of incubation temperature, δ is the effect of the interaction between body length and temperature, α is the intercept, μ is the population-specific variation in the intercept, and ε is the residual error. Both μ and ε have a mean of zero and a normal distribution.

### Multimodel averaging and inference

For all of our analyses we evaluated the relative strength of support for predictions and incorporated model uncertainty using AICc (for small sample sizes) model selection and averaging, respectively (Burnham and Anderson 2002). Average parameter estimates were calculated in the MuMIn R package (Barton 2012) using the “Natural Average” method (Grueber et al. 2011) and a 95% confidence set (all models with cumulative summed weights ≥0.95) (Burnham and Anderson 2002). We also calculated the RVI, which is the sum of the weights of all models that contain a variable. AICc values represent the trade-off between model complexity (i.e., the number of parameters) and model fit within a candidate set of models.

The effect sizes of explanatory variables in regression models are only interpretable in the units of the variable (Schielzeth 2010). In order to compare relative effect sizes we standardized our data by centering (subtracting the mean) and dividing by two standard deviations (Gelman 2008). This also allows for the interpretation of main effects for variables involved in interactions without considering the interaction because the mean for all variables is approximately zero (Schielzeth 2010).

Mixed-effects model fits were evaluated using pseudo-*R*^2^, which is the *R*^2^ between the observed versus fitted values. All variables included in each of the analyses had variance inflation factors <3, which indicated that collinearity among variables was within reasonable limits and did not substantially inflate the standard errors of our parameter estimates (Zuur et al. 2010). Diagnostics for heteroscedasticity, normality, and independence of residuals were visually inspected.

## Results

**Figure 3:**
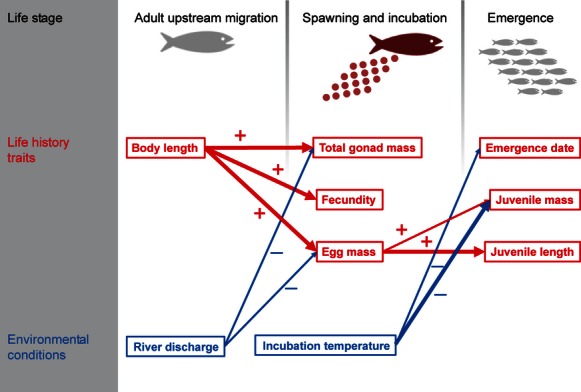
Schematic representation of the environmental (blue) and life-history (red) variables for three life stages considered in our analysis at the corresponding life stages for sockeye salmon. The thickness of arrows implies the relative strength of influence (within an analysis) and the direction of influence is denoted by positive and negative symbols (+, −).

A synthesis of the key results from the 21-year temporal analyses is in Figure 3. As predicted, females returning in years with high discharge during migration generally had lower reproductive investment. In addition, years with high incubation temperature led to smaller juveniles that emerged earlier. We also confirmed that larger females made larger reproductive investments, resulting in larger eggs and higher fecundity. Moreover, in years and creeks with larger average egg mass the resulting juveniles were also larger during out-migration. Details of these results are presented below.

### Within populations: effects of maternal length and migration difficulty on reproductive investment

**Figure 4:**
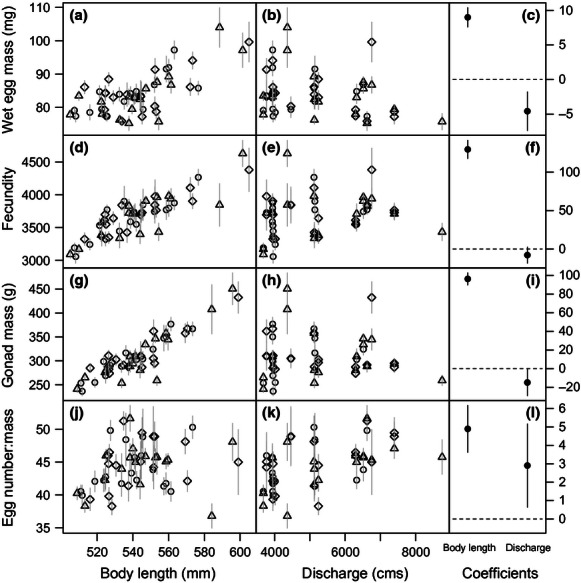
Row 1: Wet egg mass versus (a) female standard length and (b) river discharge. (c) Shows model-averaged standardized coefficients for models describing wet egg mass. Row 2: Fecundity versus (d) female standard length and (e) river discharge. (f) Shows model-averaged standardized coefficients for models describing fecundity. Row 3: Total gonad mass versus (g) female standard length and (h) river discharge. (i) Shows model-averaged standardized coefficients for models describing total gonad mass. Row 4: Ratio of egg number to egg mass versus (j) female standard length and (k) river discharge. (l) Shows model-averaged standardized coefficients for models describing the ratio of egg number to egg mass. Data are for three populations (Forfar, △ Gluskie, ○ and Kynock ♢) from 1989 to 2009. Error bars in bivariate plots are standard error and 95% confidence intervals for coefficients.

Total gonad mass decreased with migration difficulty. Contrary to our prediction, this was primarily due to production of smaller eggs, rather than reduced fecundity, resulting in higher ratios of egg number to egg mass in years when discharge was high (Fig. 4). Age at maturity and population identity had no influence on these reproductive traits (Table 1; Fig. 4).

**Table 1:** AICc 95% confidence set of models relating female body length, migration difficulty, age, and river discharge–length interaction to (A) egg mass, (B) fecundity, (C) gonad mass, and (D) the ratio of egg number to egg mass

	Reproductive trait	Model	*k*	log Lik	AICc	ΔAICc	*w*_*i*_
(A)	Wet egg mass	Discharge + Length	7	−4486.9	8988	0.00	0.70
	Wet egg mass	Age + Discharge + Length	8	−4486.8	8990	1.85	0.28
(B)	Fecundity	Age + Length	7	−9110.1	18234	0.00	0.36
	Fecundity	Length	6	−9111.3	18235	0.19	0.32
	Fecundity	Discharge + Length	7	−9110.8	18236	1.48	0.17
(C)	Gonad mass	Discharge + Length	7	−6369.5	12753	0.00	0.57
	Gonad mass	Age + Discharge + Length	8	−6369.5	12755	1.90	0.22
	Gonad mass	Length	6	−6371.9	12756	2.66	0.15
	Gonad mass	Age + Length	7	−6371.9	12758	4.67	0.06
(D)	Number:size	Discharge + Length	7	−4289.8	8594	0.00	0.55
	Number:size	Age + Discharge + Length	8	−4289.4	8595	1.16	0.31
	Number:size	Length	6	−4292.8	8598	3.84	0.08
	Number:size	Age + Length	7	−4292.1	8598	4.45	0.06

ΔAICc is the difference in AICc values between model *i* and the best model of those considered, and *w*_*i*_ is the probability that a model is the best model of the set. All models included population identity as a fixed factor and a random intercept by year.

We also confirmed the predictions that longer bodied females would have heavier gonads, heavier eggs, higher fecundity, and would produce more eggs for a given egg mass (Table 1; Fig. 4). All these reproductive traits were influenced more by body length than migration difficulty (effect sizes of body length relative to migration difficulty on: wet egg mass = 2×, fecundity = 11×, gonad mass = 8×, and egg number:size = 1.5×) (Fig. 4).

Further support that both maternal phenotype and migration difficulty shape reproductive investment comes from the results of the AIC analyses. The 95% confidence set of models describing each reproductive trait included both maternal body length (all reproductive traits, RVI = 1.0), migration difficulty (RVI range = 0.2–1.0), and in some cases age at maturity (RVI range = 0.22–0.42) (Table S2). The top models (lowest AICc values) for all response variables (Table 1) provided a good fit to our data. However, models explaining fecundity and gonad mass had much greater explanatory power than ones explaining egg size and the egg number:size ratio (pseudo *R*^2^: egg size = 0.28, fecundity = 0.50, gonad mass = 0.59, and egg number:size = 0.19).

### Within populations: effects of egg mass and incubation temperature on juvenile fitness-related traits

**Figure 5:**
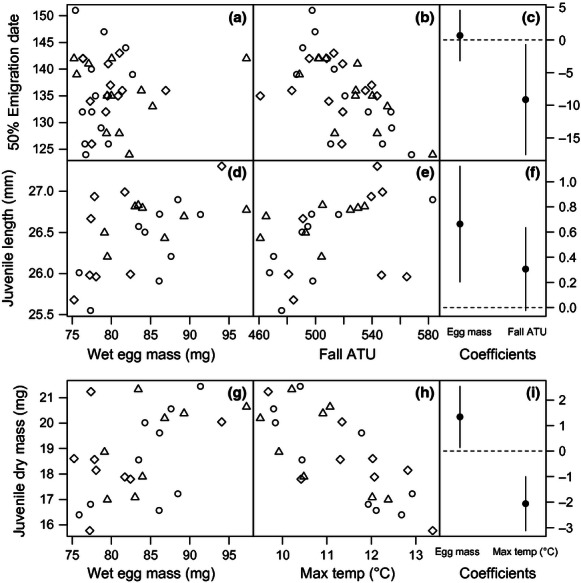
Row 1: 50% emergence date versus (a) wet egg mass and (b) Fall ATU. (c) Shows model-averaged standardized coefficients for models describing 50% emergence date. Row 2: Juvenile length versus (d) wet egg mass and (e) Fall ATU. (f) Shows model-averaged standardized coefficients for models describing juvenile length. Row 3: Juvenile dry mass versus (g) wet egg mass and (h) Max temp. (i) Shows model-averaged standardized coefficients for models describing juvenile dry mass. Data are for three populations (Forfar, △ Gluskie, ○ and Kynock ♢) from 1989 to 2009. Error bars are 95% confidence intervals for coefficients.

As predicted, warmer stream temperatures at the time of spawning (Max temp) led to lighter juveniles. This, alongside the positive effect of egg mass, explained 71% of the variation in juvenile mass (Table 2; Fig. 5). Together with egg mass, incubation temperatures explained 46% of the variation in juvenile length (Table 2). Finally, warmer fall incubation temperatures were also associated with earlier emergence, explaining a large amount of the variation in emigration date (*R*^2^ = 0.45) (Table 2).

**Table 2:** AICc 95% confidence set of models relating wet egg mass, incubation temperature, and interactions between egg mass and temperature, and population and incubation temperature to juvenile traits

	Juvenile trait	Model	*R*^2^	log Lik	AICc	ΔAICc	*w*_*i*_
(A)	Emergence	Fall ATU	0.45	−108.2	228	0.00	0.53
	Emergence	Fall ATU + Pop × Fall ATU	0.51	−106.1	230	1.78	0.22
	Emergence	Fall ATU + Egg mass	0.45	−108.1	231	2.80	0.13
	Emergence	Fall ATU + Egg mass + Pop × Fall ATU	0.51	−106.0	233	4.96	0.04
	Emergence	Fall ATU + Egg mass + Fall ATU × Egg mass	0.46	−107.7	233	5.11	0.04
(B)	Length	Fall ATU + Egg mass	0.46	−7.5	32	0.00	0.52
	Length	Egg mass	0.35	−9.7	33	0.82	0.35
	Length	Fall ATU + Egg mass + Fall ATU × Egg mass	0.46	−7.5	36	3.90	0.07
(C)	Dry mass	Max temp + Egg mass	0.71	−32.7	82	0.00	0.67
	Dry mass	Max temp	0.63	−35.7	85	2.59	0.18
	Dry mass	Max temp + Egg mass + Max temp × Egg mass	0.72	−32.4	85	3.40	0.12

ΔAICc is the difference in AICc values between model *i* and the best model of those considered and *w*_*i*_ is the probability that a model is the best model of the set. All models included population as a fixed factor. Max temp is the maximum daily mean incubation temperature and Fall ATU is the thermal units accumulated from peak spawning to 15 November.

Not surprisingly, brood years with heavier eggs produced heavier juveniles on average (Fig. 5). The effect of egg mass on juvenile length was three times greater than that of incubation temperature. The opposite was true for juvenile mass; the effect of incubation temperature on juvenile mass was 1.5× greater than that of egg mass (Fig. 4). There was little correlation between egg mass and the 50% emigration date (*R*^2^ = 0.16) (Table 2). Both incubation temperature (RVI range = 0.6–1.0) and egg mass (0.2–1.0) were included in the 95% confidence sets for all juvenile traits (Table S3).

### Among 16 populations: effect of temperature on egg mass

**Figure 6:**
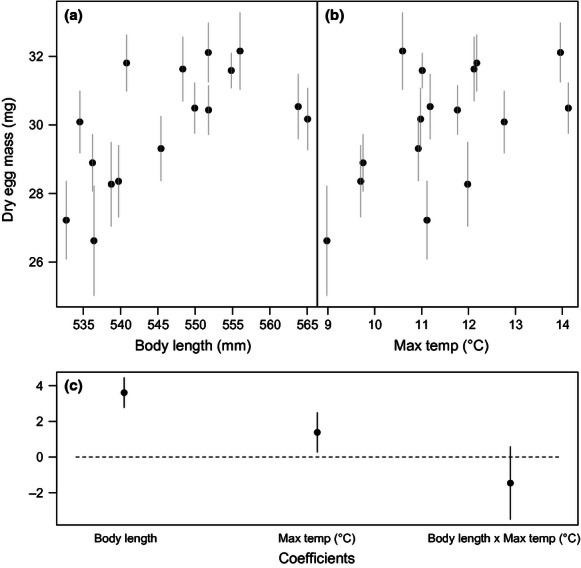
Bivariate plots of dry egg mass versus: (a) female standard length, (b) maximum daily mean incubation temperature (Max temp), and (c) a plot of model-averaged standardized coefficients for models describing dry egg size among populations. Data are for 16 populations collected in 2009. Error bars in bivariate plots are standard error and 95% confidence intervals for coefficients.

Females that spawned in streams with warmer water produced larger eggs (Table 3; Fig. 6), as predicted. This was confirmed by relating female body length, maximum daily mean incubation temperature, and a body length by temperature interaction to dry egg mass among 16 streams. As in the previous analysis, larger fish produced larger eggs (Fig. 6; Table S4). The estimated effect of female body length on egg mass was 3.3 times greater than the effect of temperature (Fig. 6). There was a strong overall fit of the top model describing dry egg mass, which included maternal body length, maximum incubation temperature, and a maternal body length by temperature interaction (pseudo *R*^2^ = 0.40).

**Table 3:** AICc 95% confidence set of models relating maternal length and maximum daily mean incubation temperature (Max temp), and a maternal length by temperature interaction to dry egg mass

Model	*k*	log Lik	AICc	ΔAICc	w_*i*_
Max temp + Length	5	−468.4	947.18	0.00	0.48
Max temp + Length + Max temp × Length	6	−467.4	947.27	0.09	0.46

ΔAICc is the difference in AICc values between model *i* and the best model of those considered, and w_*i*_ is the probability that a model is the best model of the set.

## Discussion

We found that both maternal phenotype and habitat conditions influence reproductive investment and subsequent juvenile fitness-related traits within populations. Specifically, maternal body length and annual variation in migration difficulty shaped reproductive investment, and egg mass and incubation temperatures were strongly associated with juvenile fitness-related traits based on a 21-year time series for three populations. Taken together, these results demonstrate that environmental conditions late in maturation and early in development plus maternal phenotype can influence juvenile fitness-related traits. This study also supports the hypothesis that egg mass has evolved in response to stream temperature, based on a comparison among 16 populations.

Previous studies have made comparisons among populations of correlations between migration difficulty and reproductive investment, rather than using long-term temporal comparisons that we have done here. These studies have found that populations with difficult migrations have lower reproductive investment, smaller eggs, lower fecundity, and higher ratios of fecundity to egg size (Fleming and Gross 1990; Kinnison et al. 2001; Crossin et al. 2004b). Within populations, in years of greater migration difficulty, we also found that surviving females had lower reproductive investment and smaller eggs but not lower fecundity, resulting in a higher ratio of egg number to egg mass. Two mechanisms, alone or in combination, could explain these results: (1) disproportionally higher mortality of females that invest more in reproduction or (2) facultative adjustments to energy allocated to reproduction. Previous studies offer support for both hypotheses. For instance, Patterson (2004) showed that egg mass increased consistently during migration (measured at the beginning and end of their migration) among years that varied in migration difficulty, using an alternative dataset for the same three populations. This suggests that females are unable to make facultative adjusts to egg mass during migration. In addition, these populations can suffer high levels of mortality during migration (up to 75% during high discharge) (Macdonald 2000) and on the breeding grounds due to energy depletion (Rand and Hinch 1998; Macdonald 2000; Patterson 2004), suggesting that the mortality hypothesis is a possibility. Moreover, experimental research on sockeye salmon has shown that releasing sockeye from their energetically expensive migration, through experimental manipulation of energy expenditures, does not change reproductive investment in gonad mass, egg size, or number (Patterson et al. 2004; Nadeau et al. 2009), suggesting fixed investment prior to migration. Under similar experimental conditions (i.e., migration is removed), Kinnison et al.(2001) showed that populations with more difficult migration invest more in reproduction. Contrary to our prediction, migratory difficulty influenced reproductive investment through a decrease in egg mass but not egg number, which suggests that either fecundity is set and selection occurs on high reproductive investment independent of number, or egg mass can be modestly adjusted (reduced in this case) but not egg number. Studies of Macaroni penguins (*Eudyptes chrysolophus*) have shown that the degree to which egg development overlaps with migration can cause variation in egg size between consecutively laid eggs (Crossin et al. 2010). More work is needed to distinguish between differential mortality and physiological constraints on adjusting fecundity versus egg mass.

To put the effects of migration difficulty on reproductive investment into perspective, we estimate that from low to high (two standard deviations) river discharge, females experienced approximately a 4% reduction in egg mass due to migration difficulty alone. In contrast, the estimated effects of maternal length on reproductive traits are in some cases 10 times greater than river discharge. Furthermore, the estimated average en route mortality (in addition to fishing mortality) for Early Stuart sockeye is over 30% for the same 21-year period (Cummings et al. 2011). Therefore, such small changes in egg mass due to the maternal environment will likely have little measureable influence on recruitment compared with the direct en route loss. However, the effects of migration on egg mass may combine with other impacts on reproductive output such as size-selective fishing on fecundity (Kendall et al. 2009).

Numerous studies have shown the influence of egg size on juvenile growth rate and size (Einum and Fleming 1999; Marshall and Keough 2007; Venturelli et al. 2010) and for many taxa it is well known that larger juveniles have higher survival (Beacham and Murray 1985; Sinervo 1990; Fox 1994; Williams 1994; Bernardo 1996; Chambers and Leggett 1996). In fish, juvenile size can influence swimming ability, size-selective predation (Taylor and McPhail 1985; Brännäs 1995), and the ability to endure harsh environments (Hutchings 1991). Notably, at the time of emergence there was 2.5 and eight times more variation in juvenile mass than emergence date and juvenile length, respectively (dry mass CV = 25%, emergence date CV = 10%, length CV = 4%). This suggests that for these populations mass at emergence is much more plastic than length and emergence date. The advantages of being at certain length are probably linked to burst swimming ability (Burt et al. 2011) and predator avoidance, whereas mass and emergence date likely reflect the ability to survive from emergence to the timing of zooplankton blooms. The increased metabolic cost of higher water temperatures is paid for by a reduction in mass, not length.

Our study reveals that when incubation temperatures early in development are high, juveniles emerge earlier and with lower energy reserves (i.e., lower mass) than when temperatures are cool, which could impact juvenile recruitment. Einum and Fleming (2000) showed that early emergence in Atlantic salmon (*Salmo salar*) was associated with higher survival due to a competitive advantage. Schooling sockeye salmon juveniles feed in lakes on plankton and the timing of the spring plankton bloom can be crucial. Temperatures from the previous fall do not correlate with temperatures from the following spring (unpublished data), potentially leading to mismatches in timing of juvenile emergence, energy reserves, and spring plankton bloom. Individuals that emerge with larger reserves could have an advantage over individuals with smaller reserves, especially when emerging into a food poor environment (Hutchings 1991). The advantage of larger energy reserves could also exist among populations that enter a common environment once they emerge in the spring, such as the populations in this study. Further studies of the phenology of emergence and juvenile food sources are required to better understand how potential interactions between juvenile size and emergence timing might influence recruitment.

Theory predicts a negative relationship between temperature and body size because of greater metabolic costs during development in warmer temperatures (Atkinson and Sibly 1997; Angilletta and Dunham 2003). Fleming and Gross (1990) were the first to suggest that egg mass would evolve in response to incubation temperature. Our comparison of 16 wild populations supports the hypothesis that warmer incubation temperatures select for larger eggs. These results match the relationship observed between incubation temperatures and juvenile fitness-related traits. This has consequences for maternal fitness because increases in the optimal egg mass will be traded against fecundity (i.e., warmer water – larger optimal egg mass – fewer eggs). Therefore, mothers that typically spawn in warmer streams will maximize fitness by producing larger eggs per length than mothers from cooler streams, which could lead to variation in juvenile recruitment among populations. While our results match our prediction of egg size being related to temperature, they cannot distinguish between adaptive phenotypic plasticity and evolutionary response to selection.

Alternative explanations for patterns of egg mass variation among populations of Pacific salmon have been proposed, including: juvenile growth rate (Fleming et al. 1997), migration difficulty (Kinnison et al. 2001; Crossin et al. 2004b), dissolved oxygen (Einum et al. 2002), substrate size (Quinn et al. 1995; Rollinson and Hutchings 2011), breeding competition (Fleming 1994), and hatchery supplementation within populations (Heath et al. 2003). However, a key aspect of our study is that the populations share rearing habitat and experience similar environmental conditions throughout their life cycle except during spawning and incubation (which we have measured), thus controlling for other periods in their life cycle that could also cause variation in egg size among populations, such as variation in female growth rates prior to migration (Fleming et al. 1997) and migration difficulty (Crossin et al. 2004b). Indeed, other variables not included in our analyses could affect the evolution of egg mass. We were able to test for potentially confounding effects of a few variables including dissolved oxygen, substrate size, and breeding competition on the spawning grounds and found no correlation with egg mass (Table S5). Finally, there is no hatchery supplementation for these populations. While the effect of temperature was only half as strong as the effect of maternal size, our results confirm explanations proposed in previous studies (Beacham and Murray 1990; Fleming and Gross 1990) about how temperature influences egg mass among populations. Ours is the first study to measure temperature and link it to reproductive investment and traits related to fitness of juveniles in the wild.

In conclusion, this study demonstrates how intergeneration effects of maternal phenotype and environmental conditions can accumulate, through egg mass, to influence juvenile fitness-related traits. A synthesis of these results is provided in Figure 2. Briefly, adverse environmental conditions faced by mothers during their upstream migration and by incubating embryos can lead to smaller juveniles and early emergence, a combination of circumstances that could increase their probability of starvation before zooplankton blooms in the spring. Selection for heavier eggs in warmer streams may be responsible for the observed correlation between egg mass and incubation temperature among streams. These findings bring us a step closer to understanding how the interplay between maternal traits and environmental conditions may affect maternal fitness, recruitment, and the potential evolution of egg mass.

## Supporting Information

**Data S1.** Selection of incubation temperature metrics.

**Table S1**. The relative variable importance (RVI) of incubation temperature metrics in describing juvenile length, dry mass, and 50% emigration date according to AICc. The bold values correspond to the incubation temperature metric used in the main analyses to describe juvenile traits.

**Table S2**. Multimodel-averaged coefficients for the AICc 95% confidence set of models describing (A) egg size, (B) fecundity, (C) gonad mass, and (D) the ratio of egg number to egg size. Coefficients (Coef.), unconditional standard error (SE), and 95% confidence intervals are given in standard deviation units. Standardized coefficients allow for comparisons of the relative effect size of parameters. RVI is the relative variable importance.

**Table S3.** Multimodel-averaged coefficients for the AICc 95% confidence set of models describing (A) juvenile length, (B) juvenile mass, and (C) the 50% emigration date. Coefficients (Coef.), unconditional standard error (SE), and 95% confidence intervals are given in standard deviation units. Standardized coefficients allow for comparisons of the relative effect size of parameters. RVI is the relative variable importance.

**Table S4**. Multimodel-averaged coefficients for the AICc 95% confidence set of models describing egg size among populations. Coefficients (Coef.), unconditional standard error (SE), and 95% confidence intervals are given in standard deviation units. Standardized coefficients allow for comparisons of the relative effect size of parameters. RVI is the relative variable importance.

**Table S5**. Multimodel-averaged coefficients for the AICc 95% confidence set of alternative models describing egg size among populations. Coefficients (Coef.), unconditional standard error (SE), and 95% confidence intervals are given in standard deviation units. Standardized coefficients allow for comparisons of the relative effect size of parameters. RVI is the relative variable importance. Parameters in bold indicate hypotheses examined in main analyses, nonbold parameters are alternative explanations proposed in previous studies.
