# Supplementary material for: Maternal and environmental influences on egg size and juvenile life-history traits in Pacific salmon
Source: Ecol Evol. 2013 May 8;3(6):1727–40. doi: 10.1002/ece3.555 (PMC3686205; doi:10.1002/ece3.555)
Supplement: Supplementary file 1 [file ece30003-1727-SD1.docx]

Supporting Information

*Selection of incubation temperature metrics*

We selected one of the three temperature metrics for each of the three juvenile traits by competing all combinations of explanatory variables (including wet egg mass, incubation temperature and interactions between egg mass and incubation temperature and population and temperature but only allowing models to include one of the three temperature metrics. Population was included as a fixed factor in all models to avoid pseudo-replication. From this set of models we calculated the relative variable importance (RVI) of each temperature metric for each juvenile trait and re-ran our analyses retaining only the most important temperature metric (i.e., the metric with the highest RVI).

Table S1. The relative variable importance (RVI) of incubation temperature metrics in describing juvenile length, dry mass, and 50% emigration date according to AICc. The bold values correspond to the incubation temperature metric used in the main analyses to describe juvenile traits.

|  | RVI for juvenile traits | | |
| --- | --- | --- | --- |
| Temperature metric | length | dry mass | 50% emigration date |
| Maximum daily mean incubation temperature | 0.40 | **0.99** | 0.00 |
| ATUs from peak spawning to Nov 15^th^ | **0.42** | 0.00 | **0.99** |
| ATU from peak spawning to 50% emigration date | 0.19 | 0.00 | 0.00 |

Table S2. Multi-model averaged coefficients for the AICc 95% confidence set of models describing a) egg size, b) fecundity, c) gonad mass, and d) the ratio of egg number to egg size. Coefficients (Coef.), unconditional standard error (S.E.), and 95% confidence intervals are given in standard deviation units. Standardized coefficients allow for comparisons of the relative effect size parameters. RVI is the relative variable importance.

| Analysis | Parameter | Coef. | S.E. | 95% lower CI | 95% upper CI | RVI |
| --- | --- | --- | --- | --- | --- | --- |
| Egg Size | Int | 83.44 | 3.09 | 77.39 | 89.48 | - |
|  | Age | -0.01 | 0.07 | -0.15 | 0.12 | 0.3 |
|  | Gluskie | 1.20 | 0.67 | -0.11 | 2.52 | - |
|  | Kynock | -0.68 | 0.70 | -2.05 | 0.69 | - |
|  | Discharge | -4.58 | 1.42 | -7.36 | -1.79 | 1.0 |
|  | Length | 8.99 | 0.71 | 7.60 | 10.38 | 1.0 |
| Fecundity | Int | 3747.49 | 227.38 | 3301.84 | 4193.15 | - |
|  | Age | -3.29 | 5.16 | -13.40 | 6.82 | 0.4 |
|  | Gluskie | -5.40 | 29.98 | -64.16 | 53.36 | - |
|  | Kynock | -21.19 | 31.19 | -82.32 | 39.95 | - |
|  | Discharge | 9.82 | 32.11 | -53.11 | 72.75 | 0.2 |
|  | Length | 782.78 | 36.50 | 711.24 | 854.32 | 1.0 |
| Gonad | Int | 301.65 | 13.20 | 275.77 | 327.52 | - |
|  | Age | -0.05 | 0.29 | -0.61 | 0.52 | 0.2 |
|  | Gluskie | 4.02 | 3.15 | -2.15 | 10.19 | - |
|  | Kynock | -4.43 | 3.29 | -10.88 | 2.01 | - |
|  | Discharge | -12.46 | 8.52 | -29.16 | 4.25 | 0.8 |
|  | Length | 96.24 | 3.29 | 89.79 | 102.70 | 1.0 |
| Number:Size | Int | 45.84 | 3.49 | 38.99 | 52.69 | - |
|  | Age | -0.04 | 0.08 | -0.19 | 0.12 | 0.4 |
|  | Gluskie | -0.78 | 0.57 | -1.90 | 0.34 | - |
|  | Kynock | 0.02 | 0.60 | -1.14 | 1.19 | - |
|  | Discharge | 2.50 | 1.47 | -0.38 | 5.38 | 0.9 |
|  | Length | 4.89 | 0.65 | 3.62 | 6.17 | 1.0 |

Table S3. Multi-model averaged coefficients for the AICc 95% confidence set of models describing a) juvenile length, b) juvenile mass, and c) the 50% emigration date. Coefficients (Coef.), unconditional standard error (S.E.), and 95% confidence intervals are given in standard deviation units. Standardized coefficients allow for comparisons of the relative effect size parameters. RVI is the relative variable importance.

|  | Juvenile trait | Parameter | Coef. | S.E. | 95% lower CI | 95% upper CI | RVI |
| --- | --- | --- | --- | --- | --- | --- | --- |
| a) | Length | Int | 26.54 | 0.14 | 26.26 | 26.82 | - |
|  |  | Gluskie | -0.15 | 0.19 | -0.51 | 0.22 | - |
|  |  | Kynock | 0.01 | 0.25 | -0.47 | 0.49 | - |
|  |  | Fall ATU | 0.19 | 0.20 | -0.20 | 0.58 | 0.6 |
|  |  | Egg mass | 0.66 | 0.23 | 0.20 | 1.12 | 1.0 |
|  |  | Fall ATU*Egg mass | 0.00 | 0.13 | -0.25 | 0.24 | 0.1 |
| b) | Dry mass | Int | 19.22 | 0.40 | 18.43 | 20.01 | - |
|  |  | Gluskie | -0.69 | 0.51 | -1.68 | 0.31 | - |
|  |  | Kynock | -1.18 | 0.67 | -2.48 | 0.12 | - |
|  |  | Max temp | -2.06 | 0.54 | -3.12 | -1.00 | 1.0 |
|  |  | Egg mass | 1.09 | 0.76 | -0.40 | 2.58 | 0.8 |
|  |  | Max temp*Egg mass | -0.12 | 0.65 | -1.40 | 1.16 | 0.1 |
| c) | Emigration | Int | 503.25 | 1.60 | 500.13 | 506.40 | - |
|  |  | Gluskie | -3.22 | 2.15 | -7.43 | 0.99 | - |
|  |  | Kynock | -4.74 | 2.36 | -9.36 | -0.11 | - |
|  |  | Fall ATU | -9.20 | 4.36 | -17.75 | -0.66 | 1.0 |
|  |  | Egg mass | 0.11 | 0.87 | -1.60 | 1.82 | 0.2 |
|  |  | Fall ATU*Gluskie | 1.35 | 3.85 | -6.19 | 8.89 | 0.3 |
|  |  | Fall ATU*Forfar | 2.76 | 5.38 | -7.78 | 13.30 | 0.3 |

Table S4. Multi-model averaged coefficients for the AICc 95% confidence set of models describing egg size among populations. Coefficients (Coef.), unconditional standard error (S.E.), and 95% confidence intervals are given in standard deviation units. Standardized coefficients allow for comparisons of the relative effect size parameters. RVI is the relative variable importance.

| Parameter | Coef. | S.E. | 95% lower CI | 95% upper CI | RVI |
| --- | --- | --- | --- | --- | --- |
| Int | 30.13 | 0.26 | 29.62 | 30.64 | - |
| Max temp | 1.38 | 0.56 | 0.28 | 2.48 | 1.0 |
| Length | 3.61 | 0.42 | 2.78 | 4.44 | 1.0 |
| Max temp*Length | -0.71 | 1.03 | -2.73 | 1.30 | 0.5 |

Table S5. Multi-model averaged coefficients for the AICc 95% confidence set of alternative models describing egg size among populations. Coefficients (Coef.), unconditional standard error (S.E.), and 95% confidence intervals are given in standard deviation units. Standardized coefficients allow for comparisons of the relative effect size of parameters. RVI is the relative variable importance. Parameters in bold indicate hypotheses examined in main analyses, non-bold parameters are alternative explanations proposed in previous studies.

| Parameter | Coef. | S.E. | 95% lower CI | 95% upper CI | RVI |
| --- | --- | --- | --- | --- | --- |
| Int | 30.52 | 0.68 | 29.19 | 31.85 | - |
| **Max temp** | **1.43** | **0.55** | **0.35** | **2.51** | **1.0** |
| **Length** | **3.63** | **0.42** | **2.82** | **4.45** | **1.0** |
| Female density 2005 | -0.67 | 0.52 | -1.69 | 0.34 | 0.5 |
| Dissolved Oxygen | -0.07 | 0.06 | -0.20 | 0.05 | 0.4 |
| Substrate size | 0.43 | 0.59 | -0.73 | 1.59 | 0.3 |
